# Posterior Reversible Encephalopathy Syndrome with Stroke in Puerperal Woman with High Titer of Anti-Phospholipid IgM Antibody

**DOI:** 10.1155/2018/7438676

**Published:** 2018-12-03

**Authors:** Hiroto Hirashima, Masahiro Iwamoto, Tadashi Ozawa, Shigeyoshi Kijima, Shigeki Matsubara, Akihide Ohkuchi

**Affiliations:** ^1^Department of Obstetrics and Gynecology, Jichi Medical University School of Medicine, 3311-1 Yakushiji, Shimotsuke-shi, Tochigi 329-0498, Japan; ^2^Division of Rheumatology and Clinical Immunology, Department of Medicine, Jichi Medical University School of Medicine, 3311-1 Yakushiji, Shimotsuke-shi, Tochigi 329-0498, Japan; ^3^Division of Neurology, Department of Internal Medicine, Jichi Medical University School of Medicine, 3311-1 Yakushiji, Shimotsuke-shi, Tochigi 329-0498, Japan; ^4^Department of Radiology, Jichi Medical University School of Medicine, 3311-1 Yakushiji, Shimotsuke-shi, Tochigi 329-0498, Japan

## Abstract

Posterior reversible encephalopathy syndrome with stroke is very rare in puerperal women. A 36-year-old nulliparous woman with both rheumatoid arthritis and recurrent pregnancy loss, probably due to a high titer of anti-phospholipid IgM antibody, was referred at 10 weeks of gestation. Low-dose aspirin at 100 mg/day and heparin calcium subcutaneous injection at 10,000 units/day were started before pregnancy and stopped at 35^+6^ and 40^+2^ weeks, respectively. She transabdominally delivered a male infant weighing 3,344 g at 40^+5^ weeks. A tonic-clonic seizure abruptly occurred without either hypertension or proteinuria 5 days after delivery. Intracerebral hemorrhage involving an area of 2 cm in diameter in the right frontal lobe and subarachnoid hemorrhage with PRES were confirmed. Seizure recurred 2 days after the initial episode. She showed severe headache and mild disturbance of consciousness but no neurological findings. We suggested that a high titer of anti-phospholipid IgM antibody might be associated with stroke.

## 1. Introduction

As described in the first report of posterior reversible encephalopathy syndrome (PRES) by Hinchey et al. [1], PRES is a condition that is associated with not only preeclampsia and eclampsia, which are very common in cases of PRES in pregnancy or the puerperal period, but also numerous systemic conditions in nonobstetric patients of all ages. However, PRES with intracranial hemorrhage (ICH), that is, stroke, in a pregnant woman with neither preeclampsia nor eclampsia is very rare. In addition, PRES in patients with either rheumatoid arthritis (RA) or a high titer of anti-phospholipid antibody is markedly rare; to the best of our knowledge, only 3 cases of PRES in patients with RA and 4 cases of PRES in patients positive for anti-phospholipid antibody have been reported [2–8].

Here, we present a woman with both RA and recurrent pregnancy loss probably due to a high titer of anti-phospholipid IgM antibody, but without hypertension, who suffered PRES with stroke (intracerebral and subarachnoid hemorrhage), accompanied by two tonic-clonic seizures, occurring at 5 and 7 days after delivery.

## 2. Case Report

A 36-year-old nulliparous woman with past histories of both RA and recurrent pregnancy loss (repeated abortion), probably due to a high titer of anti-phospholipid IgM antibody, was referred to our tertiary center at 10 weeks of gestation.

**Figure 1:**
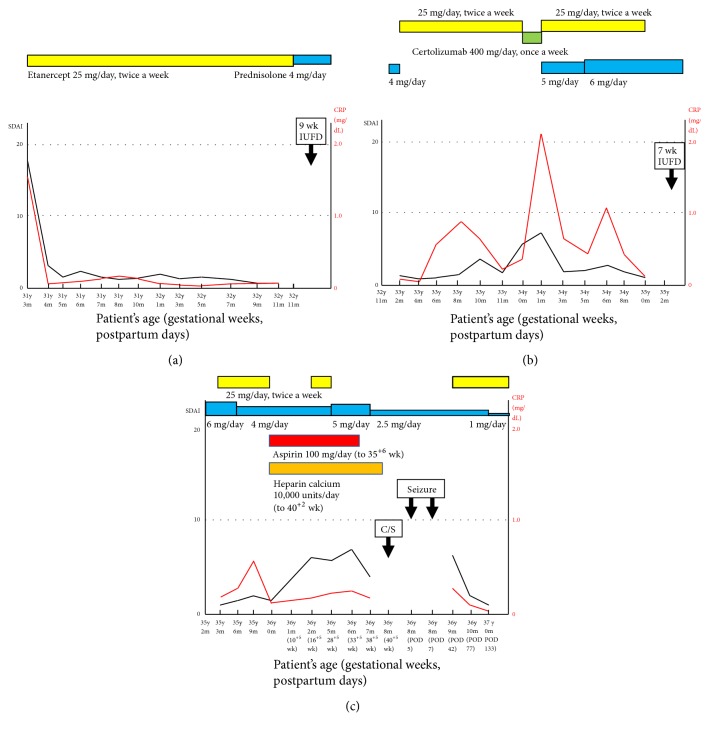
The levels of the simplified disease activity index (SDAI), which was the sum of the number of tender joints, the number of swollen joints, patient global assessment of disease activity by visual analogue scale (VAS), care provider global assessment of disease activity by VAS, and the levels of C-reactive protein (CRP) (mg/dL), and serum C-reactive protein (CRP) from the onset of rheumatoid arthritis (RA) at 31 years and 3 months old to 133 days after the current delivery. (a) The levels of SDAI and CRP from 31 years and 3 months old to 32 years and 11 months old when she became pregnant for the first time, which resulted in intrauterine fetal death (IUFD) at 9 weeks of gestation. (b) The levels of SDAI and CRP from 32 years and 11 months old to 35 years and 2 months old when she became pregnant for the second time, which also resulted in IUFD at 7 weeks of gestation. (c) The levels of SDAI and CRP from 35 years and 2 months old to postpartum day 133 in the delivery. The used drugs are shown at the top of the charts. wk, weeks of gestation; y, years; m, months; C/S, cesarean section; POD, postoperative days.

RA had been diagnosed at 31 years old, and etanercept at 25 mg/day, twice a week, was started. Within 1 month, the simplified disease activity index (SDAI) had improved from 18.1 to 2.6 (Figure 1(a)). SDAI was the sum of the number of tender joints, the number of swollen joints, patient global assessment of disease activity using a visual analogue scale (VAS), care provider global assessment of disease activity by VAS, and the level of C-reactive protein (CRP) (mg/dL). At 32 years old, she became pregnant, and etanercept was changed to prednisolone at 4 mg/day; however, intrauterine fetal death (IUFD) occurred at 9 weeks. Thereafter, prednisolone was changed to etanercept at 25 mg/day (Figure 1(b)). Since she desired to become pregnant at 35 years old, etanercept was discontinued before pregnancy; she soon became pregnant, but at 7 weeks, IUFD occurred again. Thereafter, etanercept at 25 mg/day, twice a week, was restarted with a decreased dose of prednisolone at 4 mg/day (Figure 1(c)).

Because of repeated abortion and the discovery of a high titer of anti-cardiolipin IgM antibody (twice ≥80 U/mL with intervals of ≥3 months [reference range: <8 U/mL]), low-dose aspirin at 100 mg/day and heparin calcium subcutaneous injection at 10,000 units/day were started before pregnancy, with the discontinuation of etanercept. At 36 years old, she became pregnant for a third time. Because SDAI was increased to 6.8 at 16^+5^ weeks, etanercept at 25 mg/day, twice a week, was reinitiated. However, etanercept was discontinued at 28^+5^ weeks, because the British Society of Rheumatology (BSR) and British Health Professionals in Rheumatology (BHPR) guidelines on prescribing drugs in pregnancy and breastfeeding recommended the usage of etanercept until the end of the second trimester [9]. Then, prednisolone was increased from 4 to 5 mg/day from 28^+5^ weeks; however, prednisolone was decreased from 5 to 2.5 mg/day from 38^+5^ weeks, because the SDAI was decreased from 7.9 to 4.9. Taken together, the SDAI scores during pregnancy were slightly higher than those before the current pregnancy.

**Figure 2:**
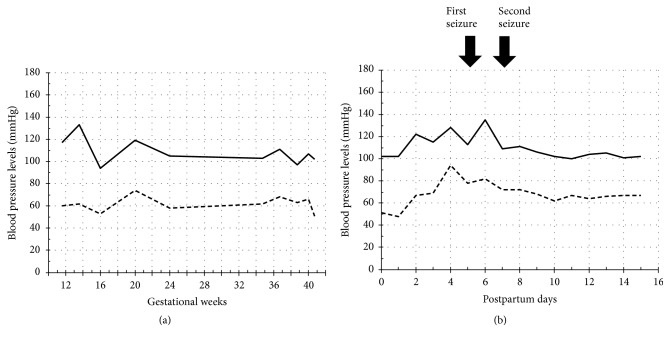
Systolic blood pressure (SBP) and diastolic blood pressure (DBP) during pregnancy and the puerperal period. (a) SBP and DBP during pregnancy. Solid jagged lines indicate SBP, whereas broken jagged lines indicate DBP. (b) SBP and DBP from delivery to 16 days after delivery. Seizure occurred 5 and 7 days after delivery.

**Figure 3:**
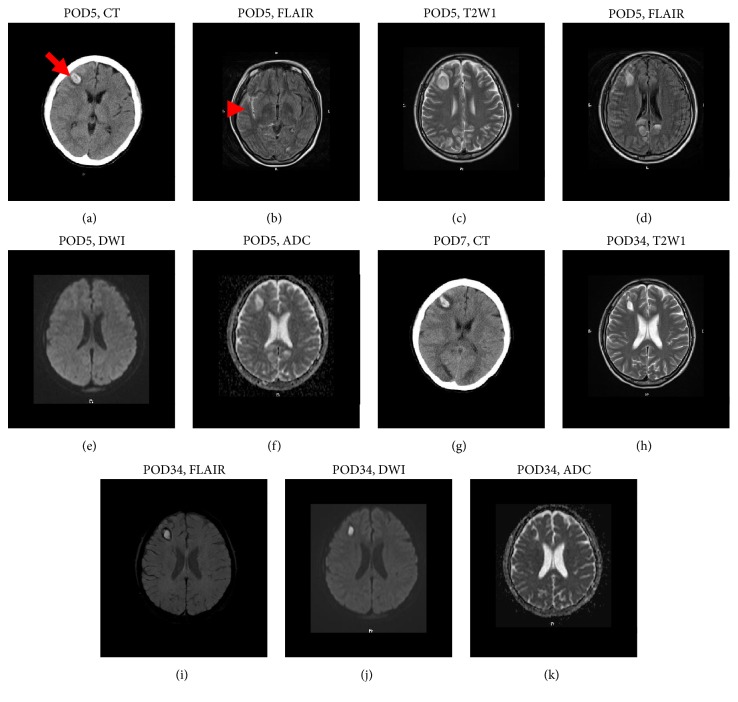
(a) CT image 5 days after delivery, just after the occurrence of tonic-clonic seizure. Arrow indicates intraparenchymal hemorrhage with 2 cm diameter in the right frontal lobe. (b) FLAIR image on MRI 5 days after delivery, just after the occurrence of tonic-clonic seizure. Arrowhead indicates subarachnoid hemorrhage at the right Sylvian fissure. (c) T2-weight image on MRI 5 days after delivery, just after the first seizure. (d) FLAIR image on MRI 5 days after delivery. (e) DWI on MRI 5 days after delivery. (f) ADC mapping on MRI 5 days after delivery. (g) CT image 7 days after delivery, just after the second tonic-clonic seizure. (h) T2-weighted image on MRI 34 days after delivery. (i) FLAIR image on MRI 34 days after delivery. (j) DWI on MRI 34 days after delivery. (k) ADC mapping on MRI 34 days after delivery. CT, computed tomography; MRI, magnetic resonance imaging; POD, postoperative days; T2W1, T2-weighted; FLAIR, fluid-attenuated inversion recovery; DWI, diffusion-weighted image; ADC, apparent diffusion coefficient.

Aspirin was stopped at 35^+6^ weeks, and heparin was stopped at 40^+2^ weeks. She transabdominally delivered a male infant weighing 3,344 g at 40^+5^ weeks due to arrest of labor following its induction. A tonic-clonic seizure abruptly occurred without either hypertension or proteinuria 5 days after delivery; her blood pressure at the first seizure was 113/78 mmHg, and that at the second seizure was 109/72 mmHg (Figure 2). Severe headache preceded the convulsion and continued after the seizure for almost 7 days. After the seizure, her consciousness was mildly disturbed (Japan Coma Scale I-3). There were no visual changes, and no hemiplegia. Computed tomography (CT) disclosed intraparenchymal hemorrhage with 2 cm diameter in the right frontal lobe (Figure 3(a)) and a fluid-attenuated inversion recovery (FLARE) image obtained by magnetic resonance imaging (MRI) disclosed subarachnoid hemorrhage at the right Sylvian fissure (Figure 3(b)). MRI also revealed PRES in the bilateral frontal, temporal, and posterior lobes (Figures 3(c)–3(f)). Laboratory data revealed that she was not complicated by either thrombocytopenia or disseminated intravascular coagulation. Levetiracetam at 1,000 mg/day, carbazochrome sodium sulfonate hydrate at 100 mg/day, and tranexamic acid at 1,000 mg/day were prescribed. Seizure recurred 2 days after the first one; however, cerebral images on CT did not change compared with those 2 days before the seizure (Figure 3(g)). MRI findings were ameliorated 1 month after the seizure (Figures 3(h)–3(k)).

We did not formally evaluate SDAI around the time of seizure occurrence; however, in retrospect, the patient reported that the patient global assessment of disease activity by VAS around the seizures was milder than in the term pregnancy period. Etanercept at 25 mg/day, twice a week, was restarted 42 days after delivery; and prednisolone was changed from 2.5 to 1.0 mg/day 133 days after delivery (Figure 1(c)). SDAI 3 months after delivery had markedly improved to 0.88.

## 3. Discussion

The PRES patient with stroke was complicated by RA. In retrospect, the patient reported that the patient global assessment of disease activity by VAS around the seizures was milder than in the term pregnancy period; thus, we suggested that activity of RA may have not been associated with the PRES and stroke. However, to the best of our knowledge, only 3 cases of RA with PRES have been reported [2–4]. These 4 cases including our case may only be the tip of the iceberg, because autoimmune disease is a well-known risk factor for the occurrence of PRES.

A high titer of anti-phospholipid IgM antibody may have been associated with PRES, although, to the best of our knowledge, only 4 cases of PRES have been reported in patients with a high titer of anti-phospholipid antibody [5–8]. Systemic lupus erythematosus (SLE) is a well-known disease that is a risk factor for the occurrence of PRES [10, 11], and SLE is sometimes accompanied by anti-phospholipid antibodies [12]. We speculate that the anti-phospholipid antibody may be related to endothelial injury and the inflammation of endothelial cells, thus, leading to PRES.

This case was very rare, showing PRES with stroke 5 days after delivery, without complication by either preeclampsia or HELLP syndrome. A high titer of anti-phospholipid antibody might have been associated with the occurrence of stroke, because the frequency of being positive for anti-phospholipid antibody in young patients with stroke has been estimated as 17.2% [13], whereas the frequency of being positive for anti-phospholipid antibody in healthy adults was around 3% [14]. These data suggest that pregnant women with a high titer of anti-phospholipid antibody might have a high risk of stroke during pregnancy and the puerperal period. In our patient, LDA and heparin were discontinued during the puerperal period.

In conclusion, a puerperal woman complicated with both RA and recurrent pregnancy loss, probably due to a high titer of anti-phospholipid IgM antibody, showed PRES with stroke 5 days after delivery, although she was not complicated with either hypertension or proteinuria. We speculate that a high titer of anti-phospholipid IgM antibody may have been associated with the occurrence of stroke. Since case reports of PRES with either RA or a high titer of anti-phospholipid antibody are still limited [2–8], the accumulation of such cases is necessary to elucidate whether RA or a high titer of anti-phospholipid antibody is a risk factor for the occurrence of PRES. In addition, from a clinical point of view, puerperal woman with a high titer of anti-phospholipid antibody may have a high risk of stroke. Therefore, the accumulation and evaluation of patients with stroke during pregnancy and the puerperal period is necessary to elucidate the role of high titer of anti-phospholipid antibody in the occurrence of cerebral vascular disease.
